# Preparation, structural characterization, bioactivities, and potential clinical applications of the polysaccharides from *Paris polyphylla*: a review

**DOI:** 10.3389/fphar.2025.1539237

**Published:** 2025-02-25

**Authors:** Ailong Sha, Yuanyuan Li

**Affiliations:** ^1^ School of Teacher Education, Chongqing Three Gorges University, Chongqing, China; ^2^ School of biology and food engineering, Chongqing Three Gorges University, Chongqing, China

**Keywords:** polysaccharides from *Paris polyphylla*, preparation, structural characterization, bioactivities, potential clinical applications, review

## Abstract

The [*Paris polyphylla* Smith var. *Yunnanensis* (Franch.) Hand. - Mazz] (The following is denoted by *P. polyphylla*) contains various chemical components such as steroidal saponins, flavonoids, polysaccharides, amino acids, etc. It is a traditional Chinese medicinal herb with important medicinal value. So far, the pharmacological research on *P. polyphylla* at home and abroad mainly focuses on compounds such as saponins and flavonoids, and there are relatively few reports on the study of *P. polyphylla* polysaccharides. In recent years, with the continuous deepening of research on the *P. polyphylla*, scientific and technological workers have gradually realized the application value of the polysaccharides from *P. polyphylla*, and the research of it has been increasing year by year. This article provides a review of the preparation, structural characterization, bioactivities, and potential clinical applications of the polysaccharides from *P. polyphylla*, aiming to provide a reference for the development, application, and further research.

## 1 Introduction

[*Paris polyphylla* Smith var. *Yunnanensis* (Franch.) Hand. - Mazz] (The following is denoted by *P. polyphylla*) is a perennial herbaceous plant belonging to the Melanthiaceae family, mainly produced in Yunnan, Guizhou, and Sichuan provinces, China. It has a slightly cold nature, small toxicity, bitter taste, and belongs to the liver meridian (Cheng et al., 2015). It is recorded in the “Pediatrics Interpretation Puzzle” and “Rihuazi Materia Medica” that *P. polyphylla* can treat pediatric convulsions, head shaking, and other neurological symptoms. It is also listed as one of the basic plants of Chinese medicinal “*P. polyphylla”* in the Chinese Pharmacopoeia (2020 Edition) and primarily contains steroid saponins, flavonoids, and polysaccharides in its roots, stem, and leaves. The traditional use has shown that *P. polyphylla* is effective against neurological symptoms such as pediatric convulsions, head shaking and epilepsy (Shen Nong’s herbal classic; Pediatrics Interpretation Puzzle; Rihuazi Materia Medica; Chinese Pharmacopoeia). *Paris polyphylla* mainly contains active ingredients such as steroidal saponins, polysaccharides, and amino acids (Chi et al., 2023), which have the effects of clearing heat, detoxifying, reducing swelling, and relieving pain. It is commonly used in clinical practice to treat heat ulcers, children’s fever, convulsions, bruises, congestion, swelling, and pain, and is a basic drug for treating insect and snake bites (Tian et al., 2024).

Previous studies have shown that the *Paris* plants have a variety of pharmacological effects and biological functions, such as anti-bacterial, antiviral, anti-inflammatory, an-tioxidant, anti-tumor, anti-aging (Tag et al., 2022). The *Paris* plants are widely used as an important ingredient in traditional folk medicine, among them, *P. polyphylla* is one of the varieties widely studied. *Paris polyphylla* polysaccharide as an important polysaccharide from Melanthiaceae plants, has also attracted the attention of researchers in recent years. Currently, numerous scholars have conducted in-depth research on its extraction process and pharmacological effects. This article reviews the preparation, structural characterization, bioactivities, and potential clinical applications of polysaccharides from *P. polyphylla*, including immunity, anti-oxidant, lipid-lowering, and liver protection, providing a reference for further research.

## 2 The preparation of *Paris polyphylla* polysaccharides

So far, methods for extracting plant polysaccharides both domestically and internationally include hot water method, acid method, alkali process, enzymolysis, ultrafiltration, microwave extraction, ultrasonic extraction, high voltage pulsed electric field method, supercritical fluid extraction, ultra high pressure extraction, subcritical water extraction, liquid phase pulse discharge method (Huang and Huang, 2020). Each extraction method has its advantages and limitations in terms of economic cost, material complexity, time consumption, environmental impact, extraction efficiency, etc. Different extraction methods can affect the structure of polysaccharides and greatly affect their biological activity and chemical properties (Niyigaba et al., 2021). So far, most domestic and foreign researchers use extraction methods for pharmacological research on plant polysaccharides, including: hot water method, microwave extraction, ultrasonic extraction, and enzymolysis. Tang et al. (2019) used the hot water method to extract polysaccharides from *Amaranthus tricolor* L., and studied *in vitro* anti-oxidant activity. Our research group used hot water extraction method in the early stage to study the anti-aging effect (Sha et al., 2022), *in vitro* anti-oxidant effect (Sha et al., 2023a), and the effects on three types of muscle movements of *Suaeda rigida* polysaccharides (Sha and Hao, 2022). Gharibzahedi et al. (2022) analyzed the *in vivo* and *in vitro* anti-tumor effects of plant polysaccharides extracted using microwave extraction and ultrasonic extraction. Wu et al. (2020) developed and optimized an efficient enzymatic hydrolysis method for the degradation of *Auricularia auricula* polysaccharides (AAP) and characterized the degradation products of AAP.

### 2.1 Optimization of extraction process of *Paris polyphylla* polysaccharides

After determining the extraction method, optimizing the extraction conditions is particularly important for the yield of *P. polyphylla* polysaccharides. Reasonable process design and parameter optimization can improve the yield of polysaccharides from *P. polyphylla,* and reduce the influence of external factors on its structure and activity (Zhao et al., 2024). Usually, single-factor optimization, orthogonal experimental design optimization, and response surface methodology optimization can be used (Yang et al., 2021; Shen et al., 2014). Single-factor experiments usually use polysaccharides yield as the evaluation index, control for a single-variable, and examine the effects of different extraction conditions on polysaccharides yield separately to determine the preliminary range of extraction variables. Orthogonal experimental design considers several factors simultaneously, seeks the best combination, and focuses on how to scientifically and reasonably arrange experiments. The response surface methodology uses multiple quadratic regression analysis to fit the relationship between variables and response values, seeking optimal process parameters. Common models include Central Composite Design (CCD) and Box Behnken Design (Wang et al., 2022; Ahmad et al., 2020). Based on single-factor experiments, both orthogonal experimental design and response surface methodology determine the optimal process conditions by considering the interactions between multiple independent variables.

The single-factor and orthogonal design were adopted by Yang et al. (2021) to optimize the extraction process of polysaccharides from leaves of *P. polyphylla*, and the results showed that the optimal extraction process was 30 min, 90°C temperature, 1: 80 solid-liquid ratio, and 3 extraction times. Under these conditions, the extraction rate was 6.55% (Table 1). The hot water extraction method was used by Shen et al. (2014) to extract polysaccharides from the leaves of *P. polyphylla*. Based on single-factor experiments, a central combination design was used to optimize the extraction conditions, the optimum conditions were as follows: extraction temperature, 90.8°C; ratio of water to raw material, 21.3: 1; and extraction time 4.8 h. Under these conditions, the experimental yield of polysaccharides was 54.18% (Table 1). From this, it can be seen that using the same extraction method and different optimization schemes for the same material results in significantly different optimal extraction processes and polysaccharides extraction rates. Compared with the orthogonal design, the central combination design requires fewer materials and has a higher polysaccharides extraction rate.

**TABLE 1:** The preparation and optimization methods of [*Paris polyphylla* Smith var. *Yunnanensis* (Franch.) Hand. - Mazz] (The following is denoted by *Paris polyphylla*) polysaccharides.

Extraction methods	Optimization methods	Solid-liquid ratio	Temperature	Time	Number of times	Extraction rate	Reference
Hot water extraction	Orthogonal design	1: 80	90°C	30 min	3	6.55%	Yang et al. (2021)
	CCD	1: 21.3	90.8°C	4.8 h	—	54.18%	Shen et al. (2014)
Ultrasound assisted extraction	Orthogonal design	1: 50	50°C	1.5 h	2	24.32 mg/g	Zhou et al. (2014)
Hot water extraction	Response surface method	1: 15	92°C	2.6 h	—	3.22%	Liu et al. (2014a), Liu et al. (2014b)
Ultrasound assisted extraction		1: 14	72°C	25 min	—	3.87%	

Three different methods (reflux extraction, hot water extraction, and ultrasound assisted extraction) were used by Zhou et al. (2014) to extract polysaccharides from the roots and stems of *P. polyphylla*. Single-factor experiments and orthogonal experiments were designed to determine the optimal extraction process conditions. The results showed that after optimization by single-factor orthogonal experimental method, the ultrasound assisted extraction method obtained the highest polysaccharides extraction rate, which was 24.32 mg/g (Table 1). The optimum extraction process was as follows: the extraction temperature was 50°C, the ratio of solid to liquid was 1: 50 (g/mL), extraction time was 1.5 h, and extraction twice. The use of reflux extraction method requires comprehensive consideration of economic costs, while its extraction rate is relatively low. Hot water extraction method is one of the most suitable methods for industrial production, with low cost and easy operation. Liu et al. (2014a), Liu et al. (2014b) used ultrasound assisted extraction and hot water extraction methods to extract polysaccharides from *P. polyphylla*. Based on single-factor experiments, response surface methodology was used to optimize the extraction conditions, and the optimal extraction conditions were obtained as follows respectively: temperature was 72°C, ultrasound power 330W, extraction for 25 min, solid-liquid ratio 1: 14 (g/mL), and temperature 92°C, extraction for 2.6 h, and solid-liquid ratio was 1: 15 (g/mL), the yield of polysaccharides was 3.87% and 3.22%, respectively (Table 1). Although both methods were optimized using response surface methodology, the optimal extraction conditions and polysaccharides yield obtained were different, which was speculated to be related to the extraction method used and the origin of the *P. polyphylla.*

### 2.2 The effects of different extraction methods of polysaccharides from Paris polyphylla on their anti-oxidant activity

The hot water extraction, ultrasound assisted extraction, and microwave-assisted extraction methods were used by Qian and Chen (2019) to extract polysaccharides from the roots and stems of *P. polyphylla*. The *in vitro* antioxidant capacities of the polysaccharides obtained by the three extraction methods were evaluated using diphenyl-2-trinitrophenylhydrazine (DPPH), hydroxyl radical (⋅OH) scavenging rate, and total antioxidant capacity. The results showed that the total antioxidant capacity of polysaccharides obtained by hot water extraction was the strongest, the polysaccharides from ultrasound assisted extraction had the strongest ability to clear ⋅OH, and the polysaccharides from microwave-assisted extraction had the strongest ability to clear DPPH free radicals. Microwave and ultrasound assisted extraction can both enhance the activities of polysaccharides in scavenging DPPH and ⋅OH, as well as improve the antioxidant activity of *P. polyphylla* polysaccharides, which is speculated to be related to changes in the composition, structure, physicochemical properties, and molecular weight of *P. polyphylla* polysaccharides. From this, it can be seen that the extraction method can affect the *in vitro* activity of *P. polyphylla* polysaccharides, and ultrasound and microwave-assisted extraction are effective ways to improve the *in vitro* antioxidant activity of *P. polyphylla* polysaccharides.

For different parts of the *P. polyphylla*, the extraction method will be different. The above ground stems and leaves of *P. polyphylla* do not contain starch (Chen et al., 2018), and the hot water extraction method can be used to maintain the integrity of the polysaccharides structure as much as possible. However, the underground roots and stems of *P. polyphylla* contain a large amount of starch, and a high extraction temperature and a long time can cause starch gelatinization, which is not conducive to polysaccharide dissolution. Therefore, the ultrasonic assisted extraction method with the advantages of fast and low temperature is selected.

Different extraction methods for *P. polyphylla* polysaccharides can affect their structure and activity, therefore, it is crucial to choose the appropriate extraction method. The use of reflux extraction method requires comprehensive consideration of economic costs, while its extraction rate is relatively low; The hot water extraction method has low cost and easy operation, making it one of the most suitable methods for industrial production and suitable for large-scale factory production; The ultrasound and microwave-assisted extraction methods have the characteristics of fast, low temperature, and high efficiency, which are suitable for the determination of polysaccharides content of *P. polyphylla,* and can be well processed in the early stage of polysaccharides extraction. Therefore, ultrasound or microwave-assisted extraction methods can be the preferred method for laboratory extraction of *P. polyphylla* polysaccharides. However, for the extraction of polysaccharides from different plants, the appropriate extraction process can be selected based on the structure, morphology, and characteristics of the plants themselves.

## 3 Structural characterization of *Paris polyphylla* polysaccharides

To analyze the structure of polysaccharides in *P. polyphylla*, it is necessary to hydrolyze the polysaccharides into monosaccharides, determine the monosaccharide composition and molecular weight, clarify the glycosidic bond connection mode of the polysaccharides through methylation reaction, measure the spectral information of the polysaccharides by UV, infrared, and optical rotation, and use 1D/2D NMR and mass spectrometry to analyze the specific structure of the polysaccharides (Pan et al., 2023). So far, the detection methods for monosaccharides mainly include thin-layer chromatography, high performance liquid chromatography (HPLC), gas chromatography, and pre-column derivatization HPLC of 1-phenyl-3-methyl-5-pyrazolone (PMP) (Zhou et al., 2019). Wang et al. (2019) used the PMP pre-column derivatization HPLC method to determine the monosaccharide composition of polysaccharides from transplanted and wild *P. polyphylla*. They tested 33 samples of wild and transplanted *P. polyphylla,* and found that the polysaccharides were mainly composed of glucose, mannose, galactose, rhamnose, and arabinose, with glucose and mannose being the main components (Wang et al., 2019). The average monosaccharide content of wild *P. polyphylla* was higher than that of transplanted *P. polyphylla*, and there was no significant difference in monosaccharide composition between wild and transplanted *P. polyphylla* from different regions (Wang et al., 2019).

**FIGURE 1:**
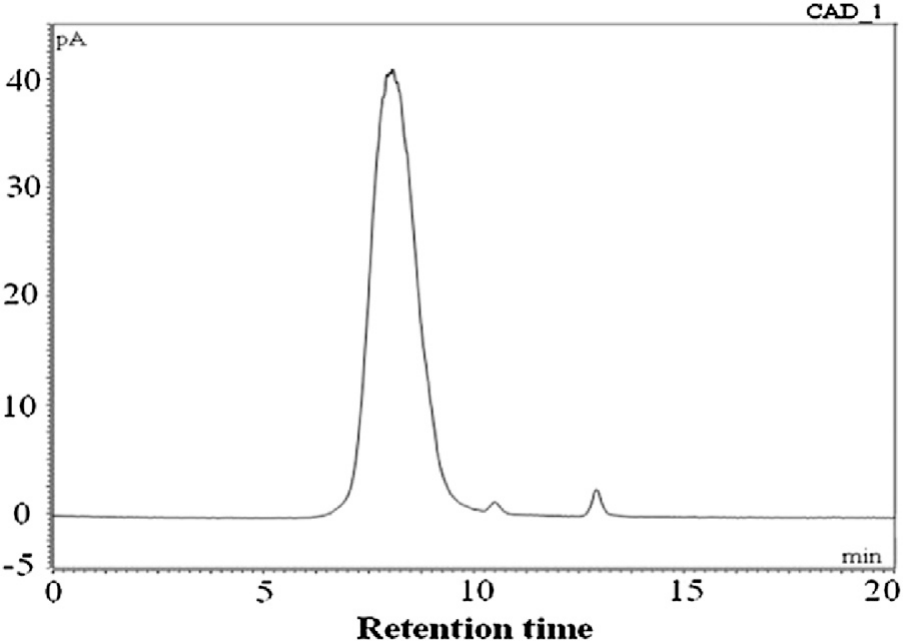
The symmetrical peak of *Paris polyphylla* polysaccharides component 1 (PPPm-1) determined by high-performance gel permeation chromatography. (Retrieved from Shen et al., 2018. Copyright 2018, International journal of biological macromolecules).

**FIGURE 2:**
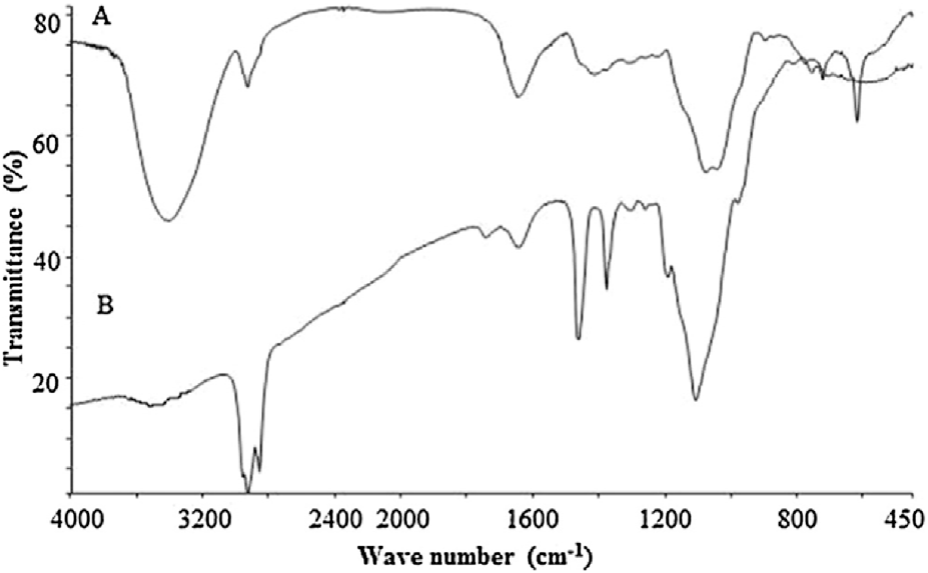
The FT-IR spectra of PPPm-1 **(A)** and methylated PPPm-2 **(B)**. (Retrieved from Shen et al., 2018. Copyright 2018, International journal of biological macromolecules).

**FIGURE 3:**
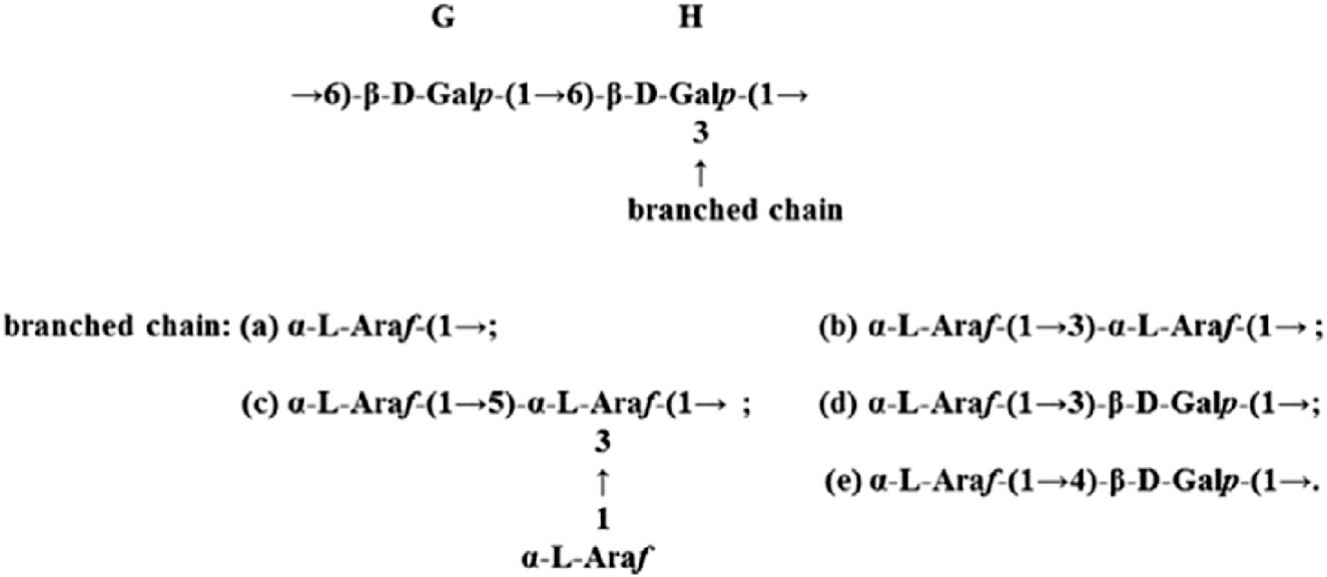
The structure of PPPm-1 (the ratio between main chain G and H was 9:31, and the ratio among branched chain a, b, c,d and e was 4:9:7:2:3). (Retrieved from Shen et al., 2018. Copyright 2018, International journal of biological macromolecules).

Shen et al. (2018) determined by gas chromatography that the *P. polyphylla* polysaccharides component 1 (PPPm-1) consisted of L-arabinose and D-galactose, with a molar ratio of 0.42: 0.58. It presented a symmetric peak in high-performance gel permeation chromatography, which indicated that PPPm-1 was a homogeneous polysaccharide (Figure 1). The retention time of PPPm-1 in gel column was 8.07 min, according to the standard curve, the average molecular weight of PPPm-1 was calculated to be 2.95 × 10^4^ Da (Shen et al., 2018). Methylation and nuclear magnetic resonance (NMR) spectroscopy data show that the main chain of PPLP is composed of 1,6- β - D-galactose, and the branch chains have five structures, mainly composed of L-arabinose residues, and are connected to the main chain through 1,3-glycosidic bonds.The FT-IR spectra of PPPm-1 (A) and methylated PPPm-2 (B) were shown in Figure 2. The broad and intense band at 3478 cm^-1^ was attributed to the ⋅OH stretching vibration (Shen et al., 2018). The absorption band at 2926 cm^−1^ was due to the stretching vibration of C-H. No absorption band at around 1730 cm^-1^ suggested that PPPm-1 did not contain uronic acid (Shen et al., 2018). The band at 1647 cm^-1^ suggested the presence of bending vibration ⋅OH. The absorption band at around 1074 cm^-1^ was assigned to the stretching vibration of C-O-C. Compared with the spectrum of PPPm-1, the absence of hydroxyl absorption band and the enhancement of C-H absorption band of methylated PPPm-1 was indicated that PPPm-1 was methylated completely (Shen et al., 2018). Comprehensive analysis of the results of methylation, partial acidolysis, and NMR, the structure of PPPm-1 was educed as shown in Figure 3, the ratio between main chain G and H was 9:31, and the ratio among branched chain a, b, c,d and e was 4:9:7:2:3 (Shen et al., 2018). Zhou et al. (2003) isolated heptose (HS) and octaose (OS) from the rhizomes of *P. polyphylla*, with molecular weights of 1152 and 1214, respectively. Theyare linear oligomers composed of glucose and mannose, the structure of HS was identified as D-Glc-(1-6)-β-D-Glc-(1-6)-β-D-Glc-(1-6)-β-D-Glc-(1-6)-β-D-Glc-(1-6)-β-D-Glc-(1-4)-α-D-Man, and OS as D-Glc-(1-6)-β-D-Glc-(1-6)-β-D-Glc-(1-6)-β-D-Glc-(1-6)-β-D-Glc-(1-6)-β-D-Glc-(1-6)-β-D-Glc-(1-4)-α-D-Man.

## 4 Bioactivities and potential clinical applications

### 4.1 Immune regulation

The spleen is an important immune organ in the human body, and the spleen index is also used as a preliminary indicator to measure the immune function of the body (Table 2). Its weight can reflect the number of lymphocytes in the immune organ to some extent, and indirectly understand the overall level of lymphocytes in the body (Xie et al., 2023). D-galactose [120 mg/(kgd)] injection was used by Liu et al. (2015) to induce an aging mouse model, and the effects of polysaccharides from *P. polyphylla* leaves (PPLP) [200 mg/(kgd)] on mouse spleen immune function were explored by detecting the mouse body weight, spleen index, and the expression levels of spleen immune gene mRNA. The results showed that compared with the model group, continuous gavage of PPLP significantly increased the body weight and the spleen index of mice (*P* < 0.05), indicating that the PPLP can improve spleen atrophy caused by D-galactose. The mRNA expression levels of T-bet, GATA-3, IL-2, IL-10, IL-4, TNF-α and INF-γ in spleen tissues of mice were significantly increased by continuous injection of D-galactose and intragastric administration of PPLP (*P* < 0.05), indicating that *P. polyphylla* polysaccharides can enhance the body’s immune function by improving humoral and cellular immunity (Liu et al., 2015). Through this study, the research reports on the immunomodulatory effects of polysaccharides from *P. polyphylla* have been supplemented, providing a scientific basis for the search and development of new safe and effective immune enhancers. However, there is currently no research report on the immunomodulatory mechanism of *P. polyphylla* polysaccharides, domestic and foreign researchers can conduct in-depth research based on this.

**TABLE 2:** Bioactivities, mechanisms, and potential clinical applications of *Paris polyphylla* polysaccharides.

Bioactivities	Action object	Related components	Action mechanism or detection index	Potential clinical applications	Reference
Immune regulation	mice	PPLP (200 mg/kg·d, purified)	Regulating the expression levels of immune related genes (T-bet, GATA-3, IL-2, IL-10, IL-4, TNF - α) and IFN - γ mRNA	Immunopotentiator	Liu et al. (2015)
Reducing blood lipid	mice	PPLP, PPRP (100, 200, 400 mg/kg·d, purified)	Increased the content of HDL-c and reduced the levels of TG, TC, and LDL-c in serum	Prevent atherosclerosis and hyperlipidemia	Wang and Sha (2020)
Anti-oxidant and Anti-aging	mice	PPLP (200 mg/kg·d, purified)	Increased T-SOD, CuZn-SOD, CAT, and GSH-Px activities and reduced MDA content	New anti-oxidants and anti-aging drugs	Shen et al. (2014), Liu et al. (2015)
	aging mice	PPLP (100, 200, 400 mg/kg·d, purified)	Enhanced anti-oxidant enzyme activity and reduced MDA content		Shen et al. (2018)
Liver protection	mice	PPLP, PPRP (100, 200, 400 mg/kg·d, purified)	Reduced MDA content, increased T-SOD and GSH-Px activities, and decreased liver index	Liver protection	Pan et al. (2023)
Enhanced learning and memory	mice	PPPm-1 (0.05, 0.1, 0.2 g/kg·d)	Inhibition of P19-P53-P21 signaling pathway, activation of Wnt/β-catenin signaling pathway, anti-oxidative stress, regulating the function of the cholinergic nervous system, enhancing LTP of long-term memory	Promoting learning and memory medications, neurotrophic healthcare products that parents and pregnant women could take during pregnancy; Treating neurodegenerative diseases	Sha et al. (2023a), Sha et al. (2024)
Muscle movement regulation	bullfrog myocardium and skeletal muscle	*Paris polyphylla* polysaccharides (purified, 1.25, 2.5, 5 mg/mL of bullfrog; 100, 200, 400 mg/kg·d of mice)	Blocking myocardial fast I_Na_ channels, inhibiting myocardial membrane I_Ca-L_, and thereby suppressing Ca^2+^ influx; enhancing the excitability of the gastrocnemius muscle in bullfrogs, increasing the number of swinging bridges, improving calcium pump activity	L-type calcium channel blocker; anti-hypertensive drugs, anti-fatigue drugs, treatment of skeletal muscle diseases (such as muscular dystrophy, myopathy, infectious myopathy, metabolic myopathy), treatment of gastrointestinal motility disorders and functional dyspepsia	Wu et al. (2021)
	mice gastrointestinal tract		Regulating gut microbiota, and affecting the release of cholinergic or adrenergic neurotransmitters at the postganglionic fiber terminals of the autonomic nervous system		Sha et al. (2023b)
Anti-fatigue	mice, frog	PPPm-1 (100, 200, 400 mg/kg·d)	Improved endurance and glycogen storage, reduced glycogen consumption, lactate, and serum urea nitrogen accumulation, promoted Ca^2+^ influx, and affected corresponding enzyme activity	Anti-fatigue	Hao and Sha, (2024)
Plant growth regulation	*Paris polyphylla, Panax ginseng*, *Nicotiana tabacum*	*Paris polyphylla* oligosaccharide (purified)	Stimulated the formation of *Paris polyphylla*’s leaves, promoted the growth of *Panax ginseng* roots and saponin accumulation, and promoted the growth of *Nicotiana tabacum* seedlings	Potential agricultural use: controlling plant growth and development	Zhou et al. (2007), Zhou et al. (2003), Liu et al. (2010)

### 4.2 Reducing blood lipid

The important indicators for judging hyperlipidemia usually include blood total cholesterol (TC), triglyceride (TG), high-density lipoprotein cholesterol (HDL-c), and low-density lipoprotein cholesterol (LDL-c), which are also the main indicators related to atherosclerosis. The lower the HDL-c content, the higher the contents of TC, TG, and LDL-c, and the more conducive to the occurrence of hyperlipidemia and atherosclerosis (Wang and Sha, 2020). Atherosclerosis index (AI) is the most reliable indicator to measure atherosclerosis. The greater AI, the more serious atherosclerosis (Ross and Harker, 1976).

*Paris polyphylla* polysaccharides affected the body weight of mice. PPLP and *P. polyphylla* rhizome polysaccharides (PPRP) could all inhibit weight gain caused by consuming high-fat feed to a certain extent without affecting the normal body weight of mice (Wang and Sha, 2020). In addition, *P. polyphylla* polysaccharides also had an impact on relevant indicators in serum. Compared with the hyperlipidemia model group, both the PPLP high-dose group and the PPRP high-dose group could significantly increase the content of HDL-c, and reduce the contents of TG, TC, and LDL-c in serum (Wang and Sha, 2020). This was consistent with the results of the effects of *Polygonatum sibiricum* polysaccharides (Ma et al., 2023), and *Astragalus membranaceus* polysaccharides (Zhao et al., 2023) on the hyperlipidemic rats. At the same time, the AI of the two groups was significantly lower than that of the hyperlipidemia model group, indicating that PPLP and PPRP had good effects in preventing atherosclerosis and hyperlipidemia.

### 4.3 Anti-oxidant and anti-aging

Oxygen reacts in the body to produce reactive oxygen species (ROS), such as ⋅OH, hydrogen peroxide (H_2_O_2_), and superoxide anions (O_2_^⋅-^). Anti-oxidation is an important process for preventing aging. Shen et al. (2014) conducted a study on the anti-oxidant activity of PPLP, and the results showed that under *in vitro* conditions, the scavenging activity of PPLP on ⋅OH, O_2_^⋅-^, and DPPH free radicals was dose-dependent, indicating that PPLP had strong antioxidant effects and was expected to become a new potential antioxidant. Liu et al. (2015) conducted *in vivo* experimental studies on the antioxidant effects of PPLP. Injecting D-galactose subcutaneously into mice, and under the action of galactose, D-galactose was broken down to produce excessive free radicals, severely damaging cells and leading to the production of lipid peroxide malondialdehyde (MDA). After continuous gavage of PPLP in mice, it was found that the activities of total superoxide dismutase (T-SOD), CuZn superoxide dismutase (CuZn-SOD), catalase (CAT), and glutathione peroxidase (GSH-Px) in the spleen were significantly increased, while the MDA content was significantly reduced (*P* < 0.05) (Liu et al., 2015). The above results indicated that the PPLP could exert its antioxidant effect by increasing the activity of antioxidant enzymes. Secondly, *P. polyphylla* polysaccharides could enhance the activity of SOD, increase the ability to scavenge free radicals, inhibit lipid peroxidation, and reduce MDA content, thereby reducing damage to body tissues and delaying aging. Shen et al. (2018) further confirmed through experiments that the PPLP exhibited strong anti-aging ability by enhancing antioxidant enzyme activity, but the anti-aging mechanism of PPLP still needed further in-depth research.

### 4.4 Liver protection

The production and removal of ROS are usually in a dynamic equilibrium in human health. However, if the body takes in too many lipid substances for a long time, the accumulation of lipids will lead to metabolic disorders, resulting in a large number of ROS. When the ROS content exceeds the clearance range of antioxidant enzymes, cell structure will be destroyed, resulting in dysfunction of body functions (Cui, 2011).

A high-fat model was established by feeding a high-fat diet to mice. It was found that compared with the normal control group, the MDA content in the liver of the high-fat model group mice increased significantly, the activities of T-SOD and GSH-Px significantly decreased, and the liver index significantly increased, indicating that high-fat diet aggravated lipid peroxidation in mice (Pan et al., 2023). The high-dose group of orally administered PPLP and PPRP significantly reduced the MDA content in the liver of mice, increased the activities of T-SOD and GSH-Px, reduced liver index, and to some extent inhibited the occurrence of lipid peroxidation in the liver, indicating that PPLP and PPRP had hepatoprotective effects (Pan et al., 2023).

### 4.5 Enhanced learning and memory

Our research group found in the early stage that the PPPm-1 can not only improve the learning and memory ability of D-galactose-induced aging model mice by inhibiting the P19-P53-P21 signaling pathway and activating the Wnt/β - catenin signaling pathway (Sha et al., 2023b), but also improve the learning and memory ability of aging pregnant mouse offspring through these two signaling pathways (Sha et al., 2024). Showing that PPPm-1 not only could provide a new intervention direction for exogenous drugs to improve learning and memory ability, but also could provide research and development directions related to neurotrophic healthcare products that parents and pregnant women could take during pregnancy to promote the neurological development of infants and adults, and improve their learning and memory abilities and intelligence (Sha et al., 2023c; Sha et al., 2024). Furthermore, our findings can also contribute to the prevention and treatment of neurodegenerative diseases (e.g., Alzheimer’s disease), thereby contributing to the field of medicine and pharmacology. However, further in-depth research is needed on the specific application of polysaccharides from *P. polyphylla* in the prevention and treatment of neurodegenerative diseases. Our previous research also found that PPPm-1 could improve D-galactose-induced learning and memory impairment in mice by antioxidant stress, regulating cholinergic nervous system function, and enhancing long-term memory (LTP) (Sha et al., 2023a). The anti-oxidant mechanism was consistent with the results of Shen et al. (2018). However, further research is needed to determine whether PPPm-1 can improve the learning and memory abilities of offspring of aging pregnant mice through the aforementioned three pathways. Our research group is also planning to conduct in-depth studies on other signaling pathways related to the two signaling pathways that enhance learning and memory abilities mentioned above, in order to carry out specific application research of *P. polyphylla* polysaccharides in the prevention and treatment of typical neurodegenerative diseases as soon as possible.

**FIGURE 4:**
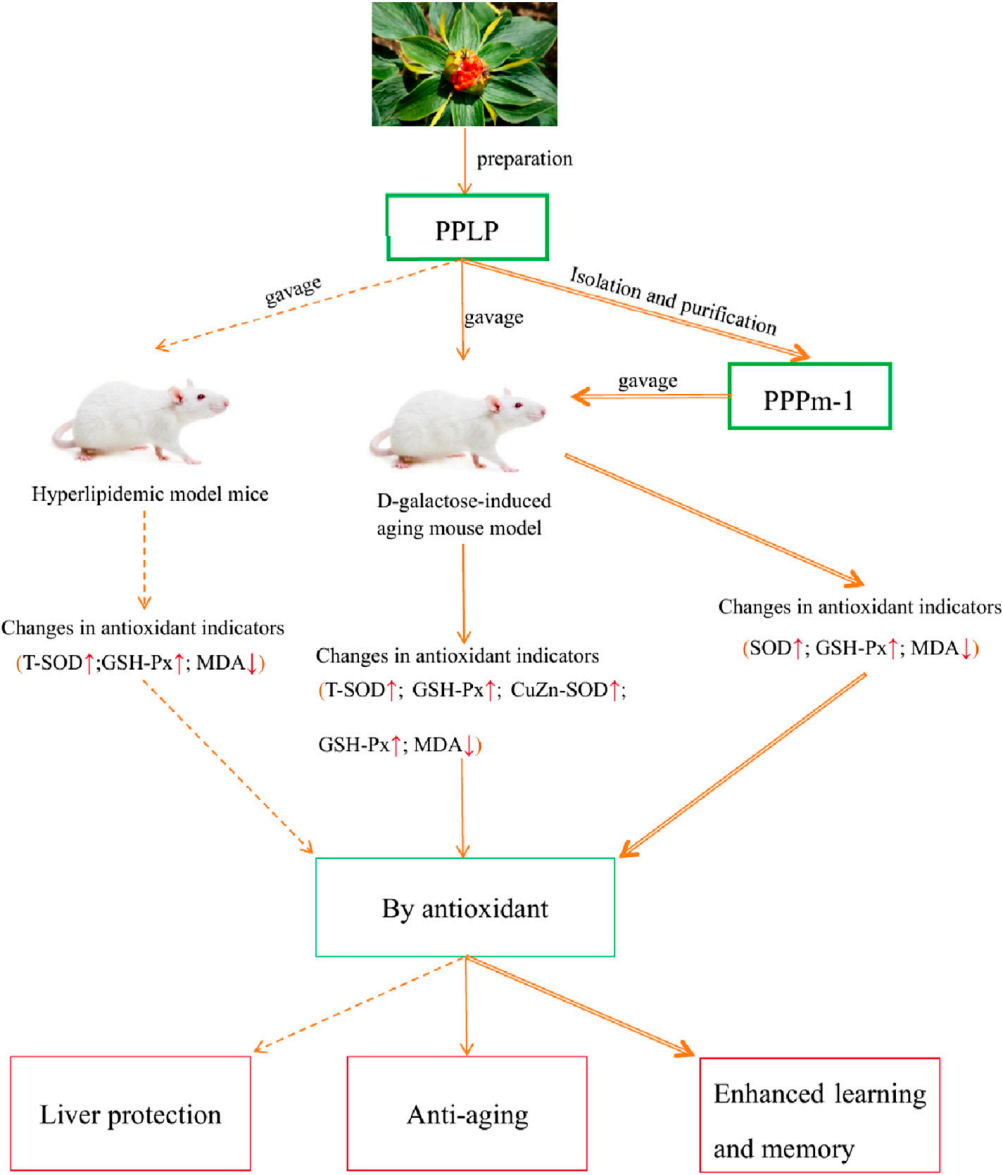
[*Paris polyphylla* Smith var. *Yunnanensis* (Franch.) Hand. - Mazz] polysaccharides achieve their anti-aging, liver protection, and learning and memory enhancing abilities through the antioxidant.

In summary, there is an inherent connection between the antioxidant effects of *P. polyphylla* polysaccharides and anti-aging, liver protection, and enhancement of learning and memory. Polysaccharides from *P. polyphylla* can delay aging by enhancing antioxidant enzyme activity and clearing free radicals; By reducing the MDA content and increasing the activities of T-SOD and GSH-Px in the liver, the occurrence of lipid peroxidation in the liver is inhibited, thereby exerting a hepatoprotective effect. It can also improve learning and memory disorders through antioxidant stress. Therefore, the mechanisms of anti-aging, liver protection, and enhancing learning and memory are all related to antioxidant effects. Polysaccharides from *P. polyphylla* can simultaneously achieve anti-aging, liver protection, and enhance learning and memory by clearing free radicals and affecting antioxidant-related enzyme activity (Figure 4).

### 4.6 Muscle movement regulation

Our research group conducted experimental studies on the effects of *P. polyphylla* polysaccharides on three types of muscle movements in animals in the early stages. The results showed that *P. polyphylla* polysaccharides had an inhibitory effect on myocardial contraction and a promoting effect on skeletal muscle contraction and relaxation of bullfrog; A low dose of polysaccharides could promote the gastrointestinal movement of mice, but a high dose could inhibit it (Wu et al., 2021). The mechanism of inhibiting myocardial contraction by *P. polyphylla* polysaccharides was related to blocking myocardial fast I_Na_ channel and inhibiting myocardial membrane I_Ca-L_, thus inhibiting Ca^2+^ inflow (Wu et al., 2021). The mechanism by which polysaccharides from *P. polyphylla* promoted the contraction and relaxation of bullfrog skeletal muscles was related to their enhanced excitability of the bullfrog gastrocnemius muscle motor unit, increased number of swinging transverse bridges, and enhanced calcium pump activity (Wu et al., 2021). The study on the effects of *P. polyphylla* polysaccharides on gastrointestinal motility in mice showed that it had a bidirectional regulatory effect on gastrointestinal motility, which might be due to its ability to regulate intestinal microbiota or affect gastrointestinal motility by affecting the release of cholinergic or adrenergic neurotransmitters at the postganglionic fiber terminals of the autonomic nervous system (Sha et al., 2023a). However, the specific mechanism of action needs further in-depth research to confirm. The above research results indicated that *P. polyphylla* polysaccharides had the potential to be used in the research and development of therapeutic drugs or health products for L-type calcium channel blockers, anti-hypertensive drugs, anti-fatigue drugs, skeletal muscle diseases (such as muscular dystrophy, myopathy, infectious myopathy, metabolic myopathy), gastrointestinal motility disorders, and functional dyspepsia, and other diseases (Wu et al., 2021; Sha et al., 2023b).

### 4.7 Anti-fatigue effect

Our research group has studied the anti-fatigue effect and mechanism of PPPm-1 in the early stage. The results showed that PPPm-1 had good anti-fatigue effects, which could significantly prolong the weight-bearing swimming time of mice (*P* < 0.01), reduce the levels of blood lactate and serum urea nitrogen in mice after exercise fatigue, and increase the levels of liver glycogen and muscle glycogen in mice (most differences are extremely significant *P* < 0.01) (Hao and Sha, 2024). 5 mg/mL PPPm-1 could significantly promote the contraction amplitude, contraction rate, and relaxation rate of frog gastrocnemius muscle, as well as the ACh content at the junction of frog sciatic nerve and gastrocnemius muscle (*P* < 0.01), but had a significant inhibitory effect on the AChE activity at the junction of frog sciatic nerve and gastrocnemius muscle (*P* < 0.01) (Hao and Sha, 2024). PPPm-1 could increase the activities of Na^+^-K^+^-ATPase and Ca^2+^-Mg^2+^-ATPase in frog gastrocnemius muscle (with *P* < 0.01 for Ca^2+^-Mg^2+^-ATPase) (Hao and Sha, 2024). The above results indicated that the main mechanisms of PPPm-1’s anti-fatigue effects were to improve endurance and glycogen reserves, reduce glycogen consumption, lactate and serum urea nitrogen accumulation, promote Ca^2+^ influx, and affect corresponding enzyme activity, suggesting that PPPm-1 can be well used for the research and development of new anti-fatigue drugs or health products.

### 4.8 Plant growth regulation

In addition to the bioactivities mentioned above, polysaccharides from *P. polyphylla* can also regulate growth. Research has shown that heptose and octose isolated from the rhizomes of *P. polyphylla* not only promoted the formation of plant buds, but also promoted the growth of *Panax ginseng* roots and the accumulation of saponins (Zhou et al., 2007; Zhou et al., 2003). Liu et al. (2010) used the polyglycosylation strategy to efficiently synthesize four oligosaccharides (pentose, hexose, heptose, and octylose) in *P. polyphylla*, and found that under different concentrations, these four oligosaccharides could stimulate the growth of *Nicotiana tabacum* seedlings to varying degrees. The above research indicates that the polysaccharides from *P. polyphylla* have good application prospects in controlling plant growth and development, but the exact mechanisms of these oligosaccharides in plant development and morphogenesis still need to be determined.

## 5 Discussion

*Paris polyphylla* is a commonly used traditional Chinese medicine in clinical practice with good medicinal value. “Diannan Materia Medica” records (Lan, 1959): “It is the best medicine for surgery, mainly used to treat all kinds of unknown swelling and toxins, and is most effective in treating various types of sores, toxins, abscesses, and boils, as well as for treating back acne and other diseases”. For a long time, the pharmacologic studies on *P. polyphylla* mainly focused on its saponins, and polysaccharides are also one of its active components, but there are few reports on the study of *P. polyphylla* polysaccharides. This article summarized the pharmacological effects of *P. polyphylla* polysaccharides, including immune regulation, reducing blood lipid, anti-oxidant and anti-aging, liver protection, enhanced learning and memory, muscle contraction regulation, anti-fatigue, and plant growth regulation (Table 2). These pharmacological effects have brought enlightenment for people to develop the medicinal value of *P. polyphylla* polysaccharides. Based on this, people can develop new immune enhancers, blood lipid-lowering drugs, antioxidants, hepatoprotective drugs, neurodegenerative drugs, L-type calcium channel blockers, anti-hypertensive drugs, anti-fatigue drugs, plant growth regulators, etc., promoting the research and application of *P. polyphylla* in the field of medicine (Table 2).

Shen et al. (2018) studied the structure and anti-aging activity of PPLP, but the structure-activity relationship between the two was less elaborated and the analysis was superficial. Therefore, more studies are needed to discover the relationship between the structure and function of *P. polyphylla* polysaccharides, and to further investigate their specific pharmacological mechanisms in one aspect. Furthermore, researchers can enhance the specific pharmacological effects or reduce adverse reactions such as toxicity and hypersensitivity reactions based on the structural modification and modification of *P. polyphylla* polysaccharides. Detailed analysis of the structures of different components of *P. polyphylla* polysaccharides (such as PPPm-1, PPPm-2) can also be conducted, and the strength of its pharmacological effects can be determined by the molecular weight of its pure components, the complexity of its structure (such as the number of branches, the position of branches, and the complexity of its spatial conformation), etc. Animal experiments can be conducted to further verify its specific pharmacological effects and efficacy.

Generally speaking, the biological activity of plant polysaccharides exhibits not only a certain structure-activity relationship, but also a certain dose-effect relationship. For example, the higher the concentration of PPPm-1, the better the effect of improving learning and memory ability (Sha et al., 2023c) and the stronger the anti-fatigue effect (Hao and Sha, 2024); The higher the concentration of PPLP, the stronger the antioxidant activity (Shen et al., 2014); *P. polyphylla* polysaccharides inhibited cardiac systole of bullfrog in a dose-dependent manner (Wu et al., 2021); There was a negative correlation between PPLP and PPRP doses and the liver index (Pan et al., 2023); The hypolipidemic effects of PPLP and PPRP were enhanced with the increase of concentration (Wang and Sha, 2020). Understanding the structure-activity and dose-effect relationship of polysaccharides is helpful for us to accurately grasp the action characteristics of *P. polyphylla* polysaccharides, so as to better develop and apply them.

As a medicinal and edible plant with multiple biological activities, *P. polyphylla* polysaccharides still have great potential for development in terms of structure and biological activity. It has great development prospects in enhancing learning and memory, anti-fatigue, muscle contraction regulation, and regulating plant growth, and is worthy of further research. In recent years, significant progress has been made in the research of *P. polyphylla* polysaccharides, but there are still some issues that cannot be ignored. Firstly, polysaccharides obtained from different raw materials and extraction optimization methods have differences in content, physicochemical properties, structural characteristics, and biological activity. Therefore, it is of great significance to establish extraction standards for *P. polyphylla* polysaccharides and develop simple and reliable quality control methods. Secondly, there is a lack of research on the pharmacokinetics of the *P. polyphylla* polysaccharides, and the studies of their transmembrane transport mode and *in vivo* process are helpful for further in-depth research on their pharmacodynamics. Thirdly, there is a lack of research on the structure-activity relationship of polysaccharides from *P. polyphylla*. It is crucial to conduct in-depth studies on the exact mechanisms and structure-activity relationships of their biological activities. This review described the structure and pharmacological effects of PPPm-1, technologists can also study other components of *P. polyphylla*, compare their structure and biological activity with PPPm-1, and analyze the structure-activity relationship of *P. polyphylla* polysaccharides. Fourthly, the current research on the *P. polyphylla* polysaccharides still remains at the cellular and animal level, but lacks clinical trials, therefore, scientific and safe clinical trials should be actively carried out.

This article reviewed the preparation, structural characteristics, bioactivities, and potential clinical applications of *P. polyphylla* polysaccharides. Different extraction methods can affect the yield and activity of polysaccharides, and the ultrasound or microwave-assisted extraction can be the preferred method for laboratory extraction of *P. polyphylla* polysaccharides. The biological activities of *P. polyphylla* polysaccharides include immune regulation, reducing blood lipid, anti-oxidant and anti-aging, liver protection, enhanced learning and memory, regulating muscle movement, anti-fatigue, and plant growth regulation. They have great application prospects in the fields of food and medicine. Based on the current research status and shortcomings of *P. polyphylla*, researchers can vigorously develop the potential value of *P. polyphylla*, in order to explore more pharmacological effects and new clinical applications of *P. polyphylla* polysaccharides.

## Abbreviations

AAP, Auricularia auricula polysaccharides; CCD, Central Composite Design; DPPH, diphenyl-2-trinitrophenylhydrazine; ⋅OH, hydroxyl radical; HPLC, high performance liquid chromatography; PMP, 1-phenyl-3-methyl-5-pyrazolone; PPLP, Paris polyphylla leaves polysaccharides; PPRP, Paris polyphylla rhizome polysaccharides; TC, blood total cholesterol; TG, triglyceride; HDL-c, high-density lipoprotein cholesterol; LDL-c, low-density lipoprotein cholesterol; PPPm-1, Paris polyphylla polysaccharide component 1; PPPm, Paris polyphylla polysaccharide m; AI, Atherosclerosis index; ROS, reactive oxygen species; H_2_O_2_, hydrogen peroxide; O_2_⋅–, superoxide anions; MDA, malondialdehyde; T-SOD, total superoxide dismutase; CuZn-SOD, CuZn superoxide dismutase; CAT, catalase; GSH-Px, glutathione peroxidase; LTP, long-term memory; Gal, galactose; Rha, rhamnose; Man, mannose; Glu, glucose; Xyl, xylose.
